# Supplementary material for: High-Throughput Label-Free Isolation of Heterogeneous Circulating Tumor Cells and CTC Clusters from Non-Small-Cell Lung Cancer Patients
Source: Cancers (Basel). 2020 Jan 3;12(1):127. doi: 10.3390/cancers12010127 (PMC7016759; doi:10.3390/cancers12010127)
Supplement: Supplementary file 1 [file cancers-12-00127-s001.pdf]

cancers MDPI

Supplementary Materials: High Throughput Label-Free Isolation of Heterogeneous Circulating Tumor Cells (CTCs) and CTC Clusters from Non-small Cell Lung Cancer (NSCLC) Patients

Isolation of CTCs from NSCLC patients (n=25) and healthy controls (n=3)

Using Labyrinth, CTCs were isolated from peripheral blood samples collected from 25 metastatic NSCLC patients along with healthy controls (n = 3). Significant differences between the overall number of CTCs in NSCLC patients samples (417±1023 per mL) vs. healthy controls (1±1.7 per mL) was observed ($p = 0.0006$). Mann-Whitney unpaired t-test analysis was used for comparing patient cohort vs. healthy control (**Figure S1**).

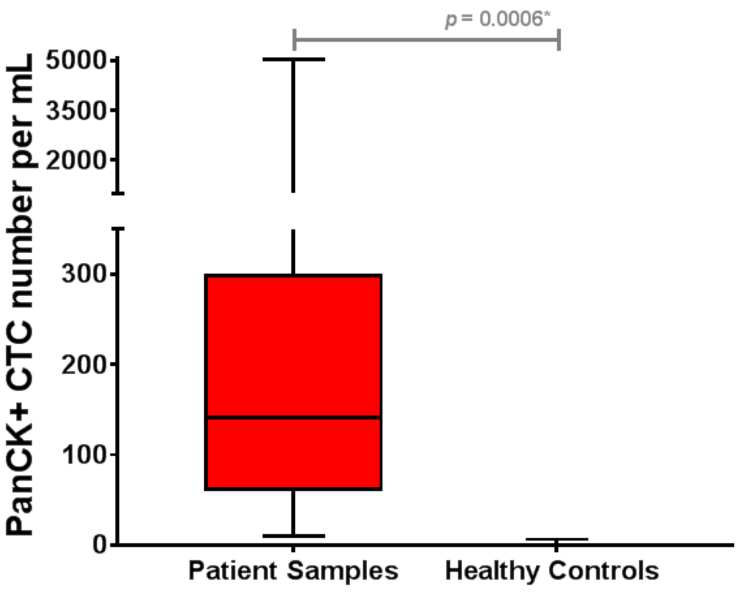

Figure S1. Comparison between the total numbers of CTCs/mL in NSCLC patients compared to healthy controls.

Identification of heterogeneous CTC/clusters subpopulations isolated metastatic NSCLC patients (n=23) using Labyrinth

Using Labyrinth, CTCs were isolated from peripheral blood samples collected from 23 metastatic NSCLC patients. Significantly higher number of EpCAM- CTCs were observed (336 CTCs/mL) compared to EpCAM+ CTCs (96 CTCs/mL) (p = 0.01). Checking the mesenchymal phenotype on the recovered CTCs demonstrated that 45% were Vimentin+ CTCs (346 CTCs/mL), and 55% were Vimentin- CTCs (85 CTCs/mL) (p = 0.4) (Figure S2A). Analyzing recovered CTC clusters from Labyrinth, we found that significantly higher numbers of CTC clusters did not express EpCAM (EpCAM-) (p = 0.005) and 41% of clusters expressed the EMT marker, Vimentin (p=0.5) (Figure S2B).

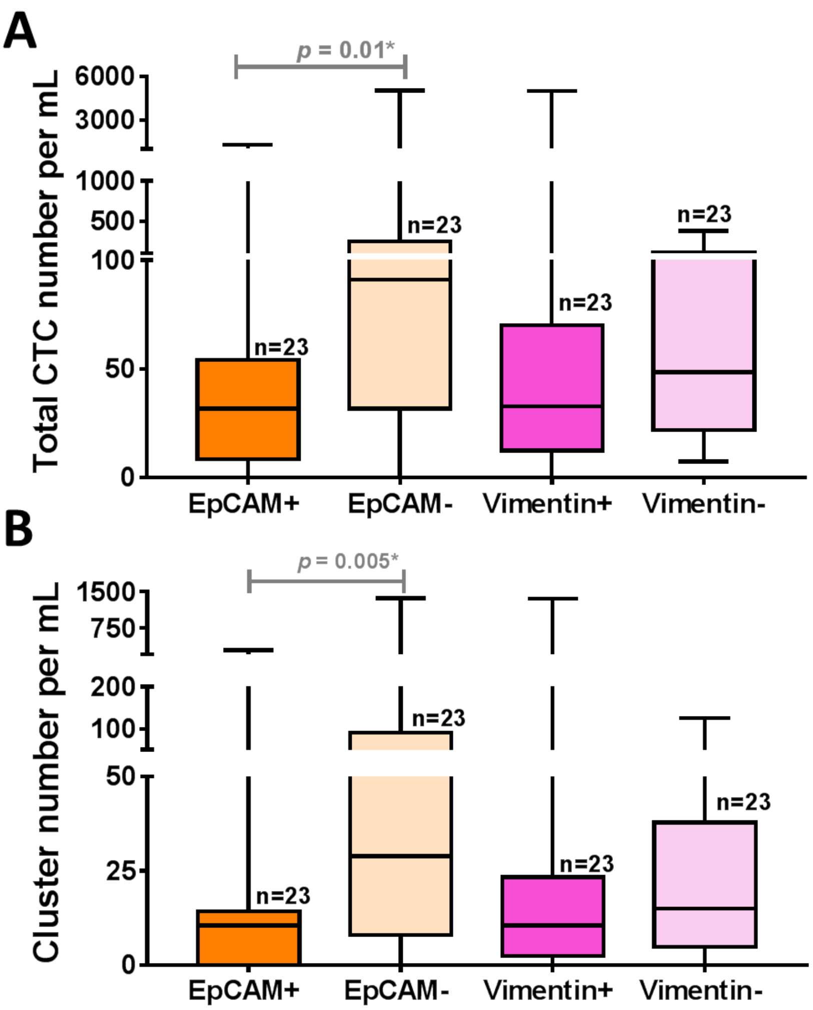

Figure S2. Analyzing heterogeneous CTC subpopulations among CTCs and CTC clusters recovered from NSCLC patients (n=23). (A) Significantly higher number of EpCAM- CTCs were observed (336 CTCs/mL) in total CTCs recovered from NSCLC patients compared to EpCAM+ CTCs (96 CTCs/mL) (p = 0.01). Checking the mesenchymal phenotype on the recovered CTCs demonstrated that 45% were Vimentin+ CTCs (346 CTCs/mL), and 55% were Vimentin- CTCs (85 CTCs/mL). (B) Analyzing recovered CTC clusters from Labyrinth, we found that significantly higher numbers of CTC clusters were not expressing EpCAM (EpCAM-) (p = 0.005), and 41% of CTC clusters expressing the EMT marker, Vimentin. Wilcoxon test analysis was used for comparing between these groups. Analyses were conducted using GraphPad Prism.

Table S1. Clinical characterization of patients with NSCLC.

Sample ID	Gender	Age	Stage	Node status	Tumor	Adenocarcinoma subtype	Smoking status	Mutation	Treatment
**P 01**	Female	39	IV	N0	T2	ALK	No	ALK mutation	Crizotinib
**P 02**	Female	55	IV	N3	T2	ROS-1	80 PY	ROS-1 mutation	Crizotinib
**P 03**	Female	40	IV	N2	T1	ROS-1	No	ROS-1 rearrangement	Crizotinib
**P 04**	Male	85	IV	N1	T2	ROS-1	30 PY	ROS-1 rearrangement	Crizotinib
**P 05**	Female	78	IV	N2	T3	EGFR	No	EGFR mutant AC with T790M mutation	Osimertinib
**P 06**	Female	49	IV	N0	T4	EGFR	25 PY	T790M mutation	Osimertinib
**P 07**	Female	77	IV	N1	T3	EGFR	No	EGFR mutant AC	Erlotinib
**P 08**	Male	81	IV	N2	T1	EGFR	No	EGFR mutant AC	Afatinib
**P 09**	Female	43	IV	N2	T2	ROS-1	No	ROS-1 mutation	Crizotinib
**P 10**	Male	58	IV	N0	T3	ROS-1	No	ROS-1 mutation	Crizotinib
**P 11**	Male	60	IV	N0	T2	ALk	23 PY	ALK translocation +	Crizotinib
**P 12**	Female	57	IV	N0	T4	EGFR	5 PY	EGFR mutation	Erlotinib and Zometa
**P 13**	Male	62	IV	N2	T3	EGFR	No	EGFR mutation	Afatinib
**P 14**	Female	58	IV	N0	T2	EGFR	5 PY	EGFR mutation	Pemetrexed
**P 15**	Female	61	IV	N0	T2	EGFR	10 PY	EGFR exon 19 deletion	Tarceva
**P 16**	Female	80	IV	N0	T2	EGFR	No	EGFR L858R, T790M	Osimertinib
**P 17**	Male	58	IV	Nx	T3	ALK	No	TTF-1, Napsin, ALK positive	Crizotanib
**P 18**	Female	61	IV	N3	T2	EGFR	No	EGFR L858R, T790M	Osimertinib
**P 19**	Male	50	IV	N2	T2	ALK	Former	ALK EML-4 fusion	Alectinib
**P 20**	Female	45	IV	N3	T4	EGFR	Former	EGFR, Exon 19 deletion, PDL1 10%	Tagrisso
**P 21**	Female	63	IV	N3	T3	ROS-1	No	ROS-1	Carboplatin/Pemetrexed
**P 22**	Male	72	IV	NA	NA	EGFR	Former	EGFR exon 19 deletion, T790M	Tagrisso
**P 23**	Male	70	IV	N0	T2a	EGFR	Former	EGFR Exon 19 deletion	Tagrisso
**P 24**	Female	70	IV	N3	T1a	EGFR	Former	EGFR Exon 19 deletion	Tagrisso
**P 25**	Male	66	IV	N0	T1a	EGFR	No	EGFR Exon 19 deletion	Tagrisso

**P 26***	Female	43	IV	N0	T2A	RET	No	RET mutation	Alectinib

NA: Not available. * Discarded from the analysis due to the different sample processing. AC: Adenocarcinoma

Table S2. CTC enumeration in patients with NSCLC.

Sample ID	CTCs	EpCAM+ CTCs	Vimentin+ CTCs	Double+ CTCs	Single CTCs	Number of CTCs in clusters	Total CTCs
**P 01**	121.4	0.0	11.3	8.5	2.8	138.4	141.2
**P 02**	34.4	0.0	7.4	54.0	27.0	68.7	95.7
**P 03**	96.9	14.2	23.6	33.1	26.0	141.8	167.8
**P 04**	100.9	38.2	30.0	2.7	65.5	106.4	171.8
**P 05**	366.7	10.0	0.0	0.0	95.4	281.3	376.7
**P 06***	177.6	NA	NA	NA	38.4	139.2	177.6
**P 07**	24.0	0.0	57.6	122.4	24.0	180.0	204.0
**P 08**	17.0	0.0	2.4	12.1	4.8	26.7	31.5
**P 09***	317.0	NA	NA	NA	34.7	282.3	317.0
**P 10**	0.0	7.5	0.0	7.5	15.1	0.0	15.1
**P 11**	331.6	0.0	0.0	31.6	47.4	315.8	363.2
**P 12**	189.3	5.2	59.6	25.9	7.8	272.2	280.0
**P 13**	31.6	0.0	0.0	7.9	21.1	18.5	39.5
**P 14**	21.8	0.0	9.5	2.7	17.7	16.4	34.1
**P 15**	42.4	4.4	0.0	5.9	30.7	22.0	52.7
**P 16**	7.3	0.0	1.5	1.5	4.4	5.8	10.2
**P 17**	71.3	0.0	11.3	56.3	97.5	41.3	138.8
**P 18**	38.5	0.0	52.5	0.0	31.5	59.5	91.0
**P 19**	6.4	6.4	22.4	35.2	16.0	54.4	70.4
**P 20**	82.5	0.0	20.6	4.1	4.1	103.1	107.3
**P 21**	41.6	6.9	263.5	239.2	20.8	530.4	551.2
**P 22**	24.0	60.0	12.0	16.0	60.0	52.0	112.0
**P 23**	17.5	0.0	336.0	1305.5	45.5	1613.5	1659.0
**P 24**	70.0	0.0	4959.5	38.5	10.5	5057.5	5068.0
**P 25**	65.3	9.3	37.3	32.7	23.3	121.3	144.7
**HC 1**	3.0	0.0	0.0	0.0	3.0	0.0	3.0
**HC 2**	0.0	0.0	0.0	0.0	0.0	0.0	0.0
**HC 3**	0.0	0.0	0.0	0.0	0.0	0.0	0.0

* Analyzed for PanCK CTC only. NA: Not available

Table S3. Single CTC and CTC cluster enumeration in patients with NSCLC.

	Single CTCs					CTC Cluster CTC >/2				
**Sample ID**	**CTCs**	**EpCAM+ CTCs**	**Vimentin+ CTCs**	**Double+ CTCs**	**Total CTCs**	**CTC Clusters**	**EpCAM+ CTC Clusters**	**Vimentin+ CTC Clusters**	**Double+ CTC Clusters**	**Total CTC Clusters**
**P 01**	0.0	0.0	2.8	0.0	2.8	48.0	0.0	2.8	2.8	53.6
**P 02**	7.4	0.0	0.0	19.6	27.0	9.8	0.0	2.5	14.7	27.0
**P 03**	18.9	2.4	0.0	4.7	26.0	33.1	4.7	7.1	9.5	54.4
**P 04**	46.4	8.2	8.2	2.7	65.5	27.3	13.6	10.9	0.0	51.8
**P 05**	95.4	0.0	0.0	0.0	95.4	120.6	5.0	0.0	0.0	125.6
**P 06***	38.4	NA	NA	NA	38.4	55.2	NA	NA	NA	55.2
**P 07**	4.8	0.0	2.4	16.8	24.0	7.2	0.0	21.6	40.8	69.6
**P 08**	2.4	0.0	2.4	0.0	4.8	4.8	0.0	0.0	2.4	7.3
**P 09***	34.7	NA	NA	NA	34.7	106.5	NA	NA	NA	106.5
**P 10**	0.0	7.5	0.0	7.5	15.1	0.0	0.0	0.0	0.0	0.0
**P 11**	36.8	0.0	0.0	10.5	47.4	110.5	0.0	0.0	10.5	121.1
**P 12**	2.6	0.0	5.2	0.0	7.8	72.6	2.6	18.1	10.4	103.7
**P 13**	13.2	0.0	0.0	7.9	21.1	7.9	0.0	0.0	0.0	7.9
**P 14**	16.4	0.0	1.4	0.0	17.7	2.7	0.0	4.1	1.4	8.2
**P 15**	20.5	4.4	0.0	5.9	30.7	10.2	0.0	0.0	0.0	10.2
**P 16**	1.5	0.0	1.5	1.5	4.4	2.9	0.0	0.0	0.0	2.9
**P 17**	37.5	0.0	11.3	48.8	97.5	15.0	0.0	0.0	3.8	18.8
**P 18**	14.0	0.0	17.5	0.0	31.5	10.5	0.0	17.5	0.0	28.0
**P 19**	6.4	0.0	0.0	9.6	16.0	0.0	3.2	6.4	9.6	19.2
**P 20**	0.0	0.0	0.0	4.1	4.1	24.8	0.0	8.3	0.0	33.0
**P 21**	3.5	0.0	0.0	17.3	20.8	17.3	3.5	83.2	58.9	162.9
**P 22**	16.0	32.0	4.0	8.0	60.0	4.0	12.0	4.0	4.0	24.0
**P 23**	3.5	0.0	3.5	38.5	45.5	3.5	0.0	101.5	294.0	399.0
**P 24**	3.5	0.0	7.0	0.0	10.5	21.0	0.0	1344.0	14.0	1379.0
**P 25**	9.3	9.3	4.7	0.0	23.3	23.3	0.0	9.3	14.0	46.7

* Analyzed for PanCK CTC only. NA: Not available.
